
JOURNAL INFORMATION
==============================
NLM Title Abbreviation: Eur Psychiatry
Journal ID: Eur Psychiatry
NLM Journal ID: 9111820
Journal ID: EPA

European Psychiatry

ISSN: 0924-9338
EISSN: 1778-3585
Publisher: Cambridge University Press

ARTICLE INFORMATION
==============================
PMCID: PMC13444206
DOI: 10.1192/j.eurpsy.2026.10183
Article ID: S0924933826101837
Article version: 1
Subjects: Front Cover (OFC, IFC) and matter

EPA volume 69 issue S1 Front cover and matter

Publication date: 2026 Jun
Electronic publication date: 2026 Jul 31
Volume: 69
Issue: Suppl 1
Issue title: Abstracts of the 34th European Congress of Psychiatry
First page: f1
Last page: f6
Copyright: © The Author(s) 2026
Copyright year: 2026
Copyright holder: The Author(s)
License: This is an Open Access article, distributed under the terms of the Creative Commons Attribution licence (http://creativecommons.org/licenses/by/4.0/), which permits unrestricted re-use, distribution, and reproduction in any medium, provided the original work is properly cited.
License URL: https://creativecommons.org/licenses/by/4.0/

Page count: 6
displayText: Special Issue
tdm-reservation: 1
tdm-policy: https://www.cambridge.org/tdm/tdmrep-policy.json

==============================

### EPA26

EUROPEAN PSYCHIATRIC ASSOCIATION

Prague, Czech Republic • 28-31 March 2026

### 34th EUROPEAN CONGRESS OF PSYCHIATRY

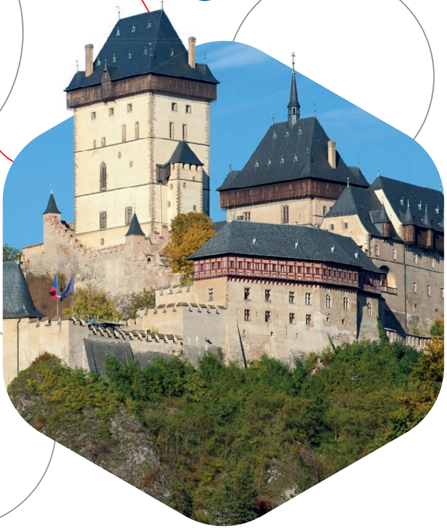

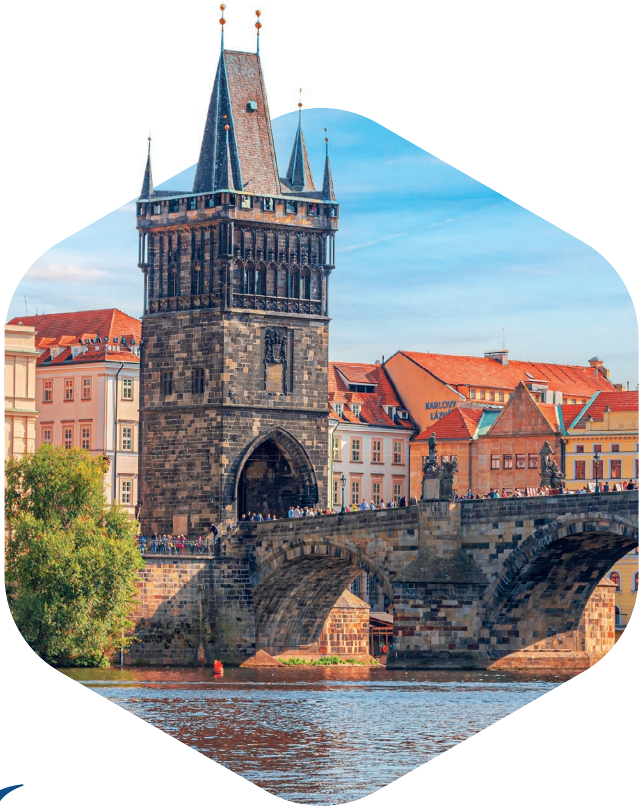

PRAGUE

### EPA 2026 Abstract Supplement

Mental Health: Improving Care and Expanding Horizons

CAMBRIDGE
UNIVERSITY PRESS & ASSESSMENT

www.epa-congress.org

#EPA2026

3 > 6
SEPTEMBER
2026

STRASBOURG
FRANCE

### EPA LEADERSHIP ACADEMY

### EMPOWERING LEADERS IN MENTAL HEALTH

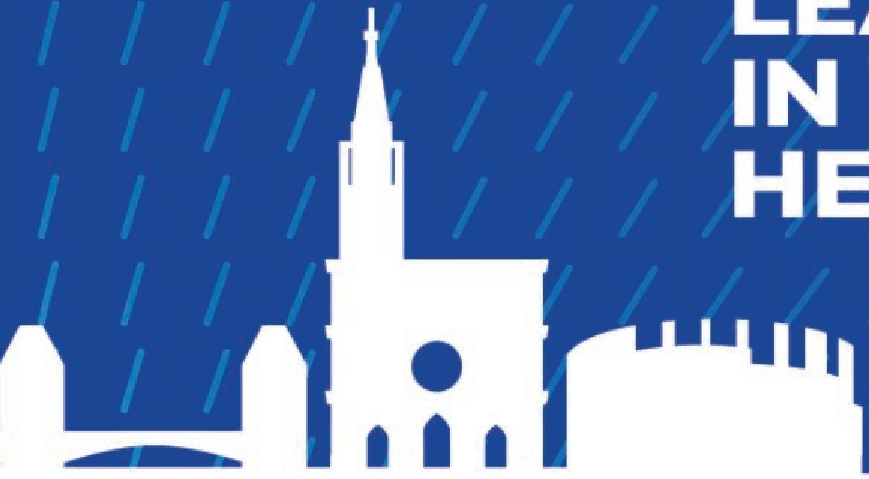

europsy.net

### EPA2027

Florence, Italy • 3-6 April 2027

EUROPEAN PSYCHIATRIC ASSOCIATION

### 35th EUROPEAN CONGRESS OF PSYCHIATRY

Innovating Mental Health:
Towards Personalised and Integrated Care

Learn more

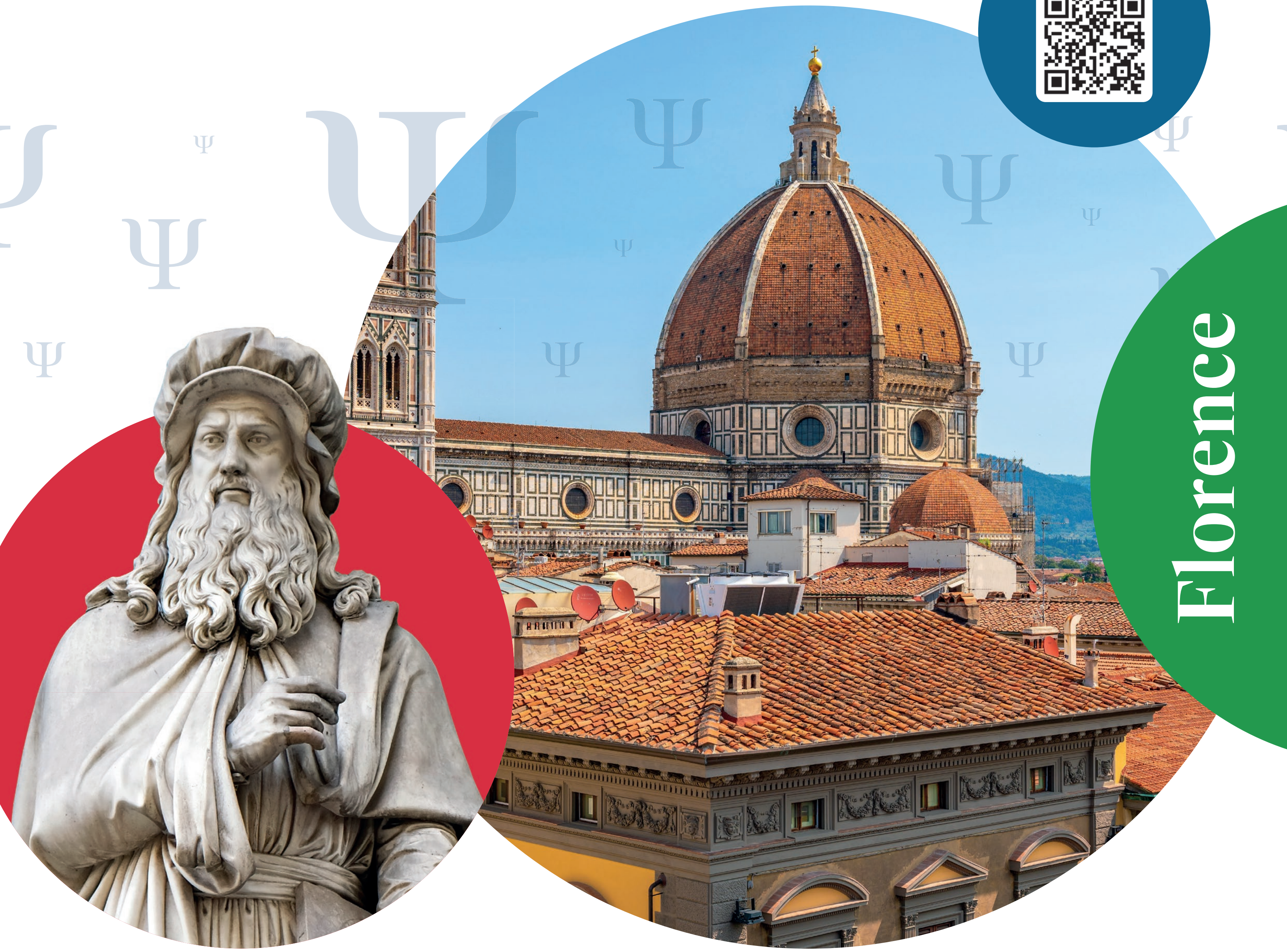

### Save the Date

#EPA2027

www.epa-congress.org

European Board Examination in Psychiatry

### Harmonising Professional Standards for Better Mental Health Care

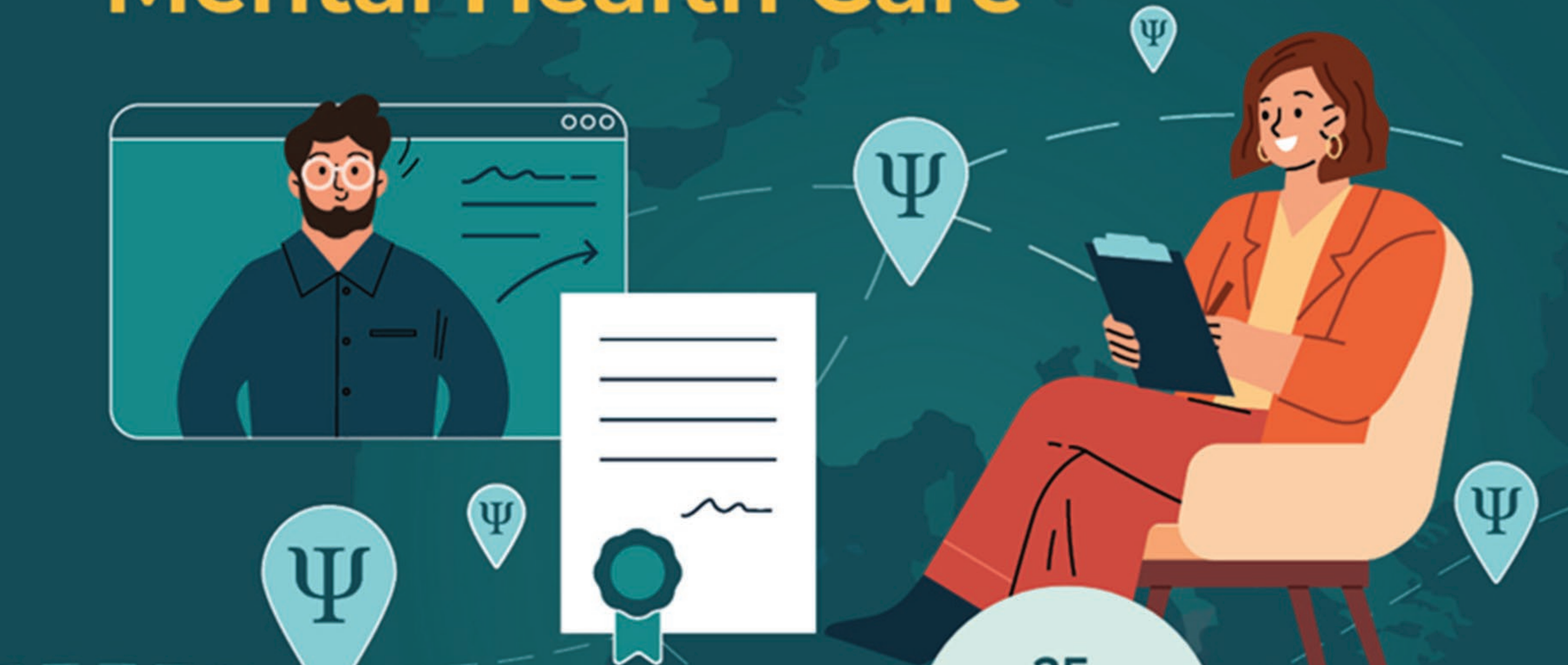

25 September 2026

APPLY NOW

europsy.net

EUROPEAN PSYCHIATRIC ASSOCIATION

**The Abstracts of the 34th European Congress of Psychiatry - 2026 are published as a Supplement to European Psychiatry. They have been peer-reviewed by the Scientific Programme Committee and the Local Organising Committee of the European Congress of Psychiatry, as well as a dedicated panel of reviewers.**

### Scientific Programme Committee (SPC)

Andrea Fiorillo, Italy – **SPC Chair**
Celso Arango, Spain – **SPC Co-Chair**
Judit Balázs, Hungary
Julian Beezhold, United Kingdom
Philippe Courtet, France
Geert Dom, Belgium
Cécile Hanon, France
Tomáš Kašpárek, Czech Republic
Pavel Mohr, Czech Republic
Martina Rojnic Kuzman, Croatia
Jerzy Samochowiec, Poland
Meryam Schouler-Ocak, Germany
Simavi Vahip, Türkiye
Umberto Volpe, Italy

### Local Organising Committee (LOC)

Tomáš Kašpárek, Brno – **Chair**
Martin Anders, Prague
Lucie Bankovska-Mottlova, Prague
Eliška Bartečková, Brno
Pavel Doubek, Prague
Iva Dudová, Prague
Michal Goetz, Opařany
Lucie Kališová, Prague
Monika Klírová, Klecany
Milan Kopeček, Klecany
Klára Látalová, Olomouc
Jiří Masopust, Hradec Králové
Petr Šilhan, Ostrava
Libor Ustohal, Brno
Jan Vevera, Plzeň
Kristina Vrbová, Olomouc

### Abstract Review Panel

Michaela Amering, Austria
Suzana Andrei, France
Gisele Apter, France
Koray Başar, Türkiye
Paolo Brambilla, Italy
Jonathan Campion, United Kingdom
Ruth Candela Ramirez, Spain
Bernardo Carpiniello, Italy
Manuel Martin Carrasco, Spain
Giovanni Castellini, Italy
His-Chung Chen, Taiwan
Eka Chkonia, Georgia
Ana Conseglieri, Spain
Marko Curak, Croatia
Marina Diaz Marsa, Spain
Paolo Enrico, Italy
Joseph Firth, United Kingdom
Sophia Frangou, United States
Fabian Friedrich, Austria
Silvana Galderisi, Italy
Thomas Gargot, France
Tomasz Gondek, Poland
Iris Graef-Calliess, Germany
Carla Maria Gramaglia, Italy
Sinan Guloksuz, Canada
David Gurrea Salas, Switzerland
Tihana Jendricko, Croatia
Helen Killaspy, United Kingdom
James Kirkbride, United Kingdom
Katja Koelkebeck, Germany
Asa Konradsson-Geuken, Sweden
Fabian Kraxner, Switzerland
Krzysztof Krysta, Poland
Tamas Kurimay, Hungary
Federico Mucci, Italy
Matilda Naesström, Sweden
Merete Nordentoft, Denmark
Jesper Norgaard Kjaer, Denmark
Alessandro Pigoni, Italy
Martin Preisig, Switzerland
Pilar Saiz, Spain
Gaia Sampogna, Italy
Norman Sartorius, Switzerland
Elisabetta Scanferla, France
Frauke Schultze-Lutter, Germany
Asilay Seker, United Kingdom
Liene Sile, Latvia
George Stercu, Romania
Anne-Laure Sutter, France
Anna Szczegielniak, Poland
Livio Tarchi, Italy
Karine Tataryan, Armenia
Agata Todzia-Kornas, Poland
Caroline Vandeleur, Switzerland
Jen-Pang Wang, United Kingdom
Danuta Wasserman, Sweden
Dora Wynchank, Netherlands

*European Psychiatry* (ISSN 1778-3585 (Online)) 2026 (volume 69). Published by Cambridge University Press.

### Abstract author inquiries

For inquiries please contact Sarah Maddox: sarah.maddox@cambridge.org

Contents of the Supplement are available on the journal website:

https://www.cambridge.org/core/journals/european-psychiatry

### The Journal of the European Psychiatric Association

EPA Membership (120 €) includes free access to the *European Psychiatry* Archives. If you are interested in becoming a member of EPA, please visit https://www.europsy.net/become-a-member

CAMBRIDGE UNIVERSITY PRESS

Vol. 69, Supplement March 2026

CONTENTS

EUROPEAN PSYCHIATRIC ASSOCIATION

Abstracted in: Science Citation Index (SCI) - Clarivate, Social Science Citation Index (SSCI) - Clarivate, Scopus, Medline/PubMed, Google Scholar, PsycINFO

### Abstracts of the 34th European Congress of Psychiatry - 2026

Plenary ........ S1
State of the Art ........ S2
Symposium ........ S4
Workshop ........ S39
ECP Programme ........ S54
Oral Communication ........ S62
e-Poster Presentation ........ S121
e-Poster Viewing ........ S418
